# Chronic wound assessment and infection detection method

**DOI:** 10.1186/s12911-019-0813-0

**Published:** 2019-05-24

**Authors:** Jui-Tse Hsu, Yung-Wei Chen, Te-Wei Ho, Hao-Chih Tai, Jin-Ming Wu, Hsin-Yun Sun, Chi-Sheng Hung, Yi-Chong Zeng, Sy-Yen Kuo, Feipei Lai

**Affiliations:** 1 Graduate Institute of Biomedical Electronics and Bioinformatics, National Taiwan University, Room 410, Barry Lam Hall, No.1, Sec.4, Roosevelt Road, Taipei, 10617 Taiwan, Republic of China; 2 Department of Surgery, National Taiwan University Hospital, No.1, Changde St., Zhongzheng Dist., Taipei, 10048 Taiwan, Republic of China; 3 Department of Internal Medicine, National Taiwan University Hospital, No.1, Changde St., Zhongzheng Dist., Taipei, 10048 Taiwan, Republic of China; 4 Data Analytics Technology and Applications Research Institute, Institute for Information Industry, 11F, No. 106, Sec. 2, Heping E. Rd., Taipei, 106 Taiwan, Republic of China; 5 Department of Electrical Engineering, National Taiwan University, No.1, Sec. 4, Roosevelt Road, Taipei, 10617 Taiwan, Republic of China

**Keywords:** Clustering, Edge detection, Image segmentation, Machine learning, Medical image processing, Surgical site classification, Wound assessment

## Abstract

**Background:**

Numerous patients suffer from chronic wounds and wound infections nowadays. Until now, the care for wounds after surgery still remain a tedious and challenging work for the medical personnel and patients. As a result, with the help of the hand-held mobile devices, there is high demand for the development of a series of algorithms and related methods for wound infection early detection and wound self monitoring.

**Methods:**

This research proposed an automated way to perform (1) wound image segmentation and (2) wound infection assessment after surgical operations. The first part describes an edge-based self-adaptive threshold detection image segmentation method to exclude nonwounded areas from the original images. The second part describes a wound infection assessment method based on machine learning approach. In this method, the extraction of feature points from the suture area and an optimal clustering method based on unimodal Rosin threshold algorithm that divides feature points into clusters are introduced. These clusters are then merged into several regions of interest (ROIs), each of which is regarded as a suture site. Notably, a support vector machine (SVM) can automatically interpret infections on these detected suture site.

**Results:**

For (1) wound image segmentation, boundary-based evaluation were applied on 100 images with gold standard set up by three physicians. Overall, it achieves 76.44% true positive rate and 89.04% accuracy value. For (2) wound infection assessment, the results from a retrospective study using confirmed wound pictures from three physicians for the following four symptoms are presented: (1) Swelling, (2) Granulation, (3) Infection, and (4) Tissue Necrosis. Through cross-validation of 134 wound images, for anomaly detection, our classifiers achieved 87.31% accuracy value; for symptom assessment, our classifiers achieved 83.58% accuracy value.

**Conclusions:**

This augmentation mechanism has been demonstrated reliable enough to reduce the need for face-to-face diagnoses. To facilitate the use of this method and analytical framework, an automatic wound interpretation app and an accompanying website were developed.

**Trial registration:**

201505164RIND, 201803108RSB.

## Background

TODAY, numerous patients suffer from chronic wounds and wound infections. As reported, the population prevalence rate of chronic wounds in the United States is roughly 2% of general population and 8.5% of the elders. The cost of treatment is about $25 billion per year [1]. With the growing demand for more efficient wound care after surgery, the development of information technology to assist the work of medical personnel has become a major trend to address these types of problems and reduce the costs of chronic wound care.

The current methods employed to solve this type of problem include: Dini et al. [2] use infrared photography to interpret wound temperature changes; Lubeley et al. [3] propose mobile three-dimensional (3D) wound measurement; Hani et al. [4] perform 3D surface scans of woundcs to obtain wound top area, true surface area, depth, and volume; Wannous et al. [5] develop imaging methods with depth of field information to judge the depths of wounds. However, these methods are expensive and require special photographic equipment; therefore, they cannot be widely used on general surgery patients.

There are already several commercial software on the market for clinicians to do wound measurement. All the software; however, has yet to incorporate automated or semi-automated wound detection or segmentation. For example, Wendelken et al. [6] measure wounds by calculating wound areas; PictZar Digital Planimetry Software [7] is a commercial software for wound analysis which provides measurements such as length, width, surface area, circumference, and estimated volume to the users. But these software require user drawings and calibration to carry out the above measurements.

To minimize the clinician’s initial involvement. Major trends toward solving this type of problem are to design special algorithms to automatically recognize the wound area then analyze the texture characteristics and color changes of the wound. But the outcome of this approach are strongly correlated to the segmentation result. This is due to background and noise in the original captured images. For example, Oduncu et al. [8] use hue, saturation, and intensity to measure the color changes of chronic wounds on the skin, but this method cannot determine the type of wound; Plassmann et al. [9] propose two active contour models to measure leg ulcers, but this method cannot assess whether the wound is infected; Kosmopoulos et al. [10] apply digital analysis to classify regions appearing in pressure ulcer images, but this method can not be widely applied to other wound types; Hettiarachchi et al. [11] attempt wound segmentation and measurement in a mobile setting based on active contour models which identifies the wound border irrespective of coloration and shape, but the method is rather sensitive to camera distance, angle and lighting conditions; Zheng et al. [12] present a new tissue classification protocol using the RGB histogram distributions of pixel values from wound color images, but this method cannot locate wound sites; Mukherjee et al. [13] propose the framework for automated tissue classification to assist the clinicians to estimate wound healing progression. Bayesian and support vector machine (SVM) were trained by using color and textural features for classifying granulation, slough, and necrotic tissues. But their methods were confined to the small dataset of images acquired under ideal imaging conditions.

Therefore, there is a high demand for developing practical, robust wound segmentation methods suitable for most lighting conditions, captured size or angles, and a series of machine learing based wound assessment algorithms to auto locate wound sites then automate the wound healing tracking process.

This paper has two major parts: (1) robust wound image segmentation, and (2) SVM-based wound infection assessment. In the first part, an edge- and color-based self-adaptive threshold detection image segmentation algorithm is proposed to filter out unnecessary background noise information. In this algorithm, robust edge detection is applied to wound images to enforce detected edges as strong edges. The skin area image is thus established by excluding pixels that are not skin-colored and normalized by the appropriate skin color values.

From our observation, the delineated wound area is correlated to the detected edges of the skin area. Therefore the edge detector must calculate candidate and optimized threshold values; this is done by considering skin wrinkles to avoid incorrect threshold adjustments. Finally, a complementary operation is performed to reconstruct the wound area from the original image.

In the second part, we propose an algorithm to position wound suture site using feature points extracted by morphological cross-shaped features from the wound area. Then we propose an optimal clustering method based on a unimodal Rosin threshold algorithm to decide the optimal clustering number. The corresponding feature point sets and ROIs are obtained from the specified optimal clustering number.

In each ROI, we calculate the corresponding feature vectors for each symptom. These feature vectors are trained by a SVM-based wound infection assessment module for wound infection decision-making support. The following symptoms can be detected by this infection detecting module: (1) Swelling, (2) Granulation, (3) Infection, and (4) Tissue Necrosis.

The assessment results were validated by a retrospective study using confirmed wound pictures from three surgical physicians. The statistics and comparison for the agreement among three physicians are presented in the later section. The assessment results for 134 wound images indicate our method achieve high sensitivity with low false positive and low false negative rates for a wide variety of wound sites and image capture lighting conditions.

Throughout the modern world, advances in mobile technology have made taking and uploading photos to the cloud a daily part of life. In that context, an automatic wound interpretation app and its accompanying website are developed to help automate wound healing tracking; this app can also aid patients for wound self-monitoring.

The remainder of this paper is organized as follows. In Methods section, we present the methods for robust image segmentation and SVM-based wound analysis interpretation in detail; we also describe the practical applications of each method in this part. Then the results are presented in Results section. Finally, conclusions are offered in Conclusion section.

## Methods

### Collect wound materials

In this experiment, total 293 wound images in use are provided by Department of Surgery and Department of Internal Medicine of National Taiwan University Hospital (NTUH), with Institutional Review Board (IRB) approval. Participants gave consent for these photos to be taken. And gave written consent for these images to be published. These wound images were captured using below two phone manufacturers (Apple iPhone 6 plus and Samsung Galaxy S6) under different settings and capture conditions. This could simulate the variation that we expect to see in terms of patient variability, wound type variability as well as variation due to image capture. This data set is split into two sets composed of 159 training data and 134 testing data. The detailed description of this data set can be found in the later section.

### Robust wound image segmentation

From our literature review, recent works for wound segmentation include: Song and Sacan [14] apply neural networks, k-means clustering, edge detection, thresholding and region growing to do wound segmentation for foot ulcers images (78 training, 14 testing). It achieves 71.4% accuracy (MLP kernel) and 85.7% accuracy (RBF kernel); Wantanajittikul et al. [15] apply FCM & morphology, texture analysis and SVM to do image segmentation and characterization for 5 images (burn cases). It achieves 72.0–98.0% accuracy; Hani et al. [16] apply ICA and k-means to do granulation detection and segmentation for 30 wound region images. It achieves 88.2% sensitivity 98.8% specificity; Veredas et al. [17] apply mean shift & region growing to do wound segmentation and tissue characterization for 113 wound region images; Hettiarachchi et al. [11] apply active contour to do wound segmentation for 20 wound region images under controlled conditions, it achieves 90.0% accuracy; Wannous et al. [18] apply mean shift, JSEG, CSC & SVM to do wound segmentation for 25 images with background under controlled conditions. It achieves 73.3–80.2% (granulation), 56.4–69.8% (slough), 64.9–70.7% (necrosis).

#### Nonwounded area suppression

##### Robust edge detection and enforcement

**Fig. 1:**
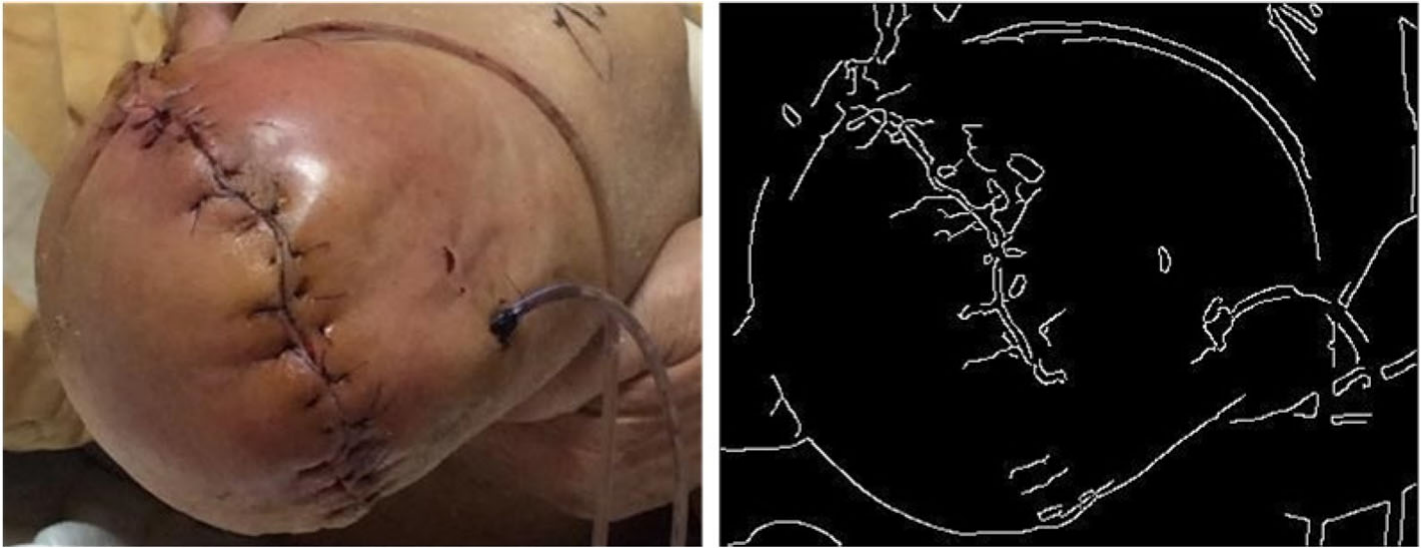
Original wound image and the edge detected result after applying the CED

From out observation, the wound region is identified by edges in the skin area. So we apply Canny edge detection [19] to distinguish the edges in the examined image. The original image and the detected edges are depicted in Fig. 1.

**Fig. 2:**
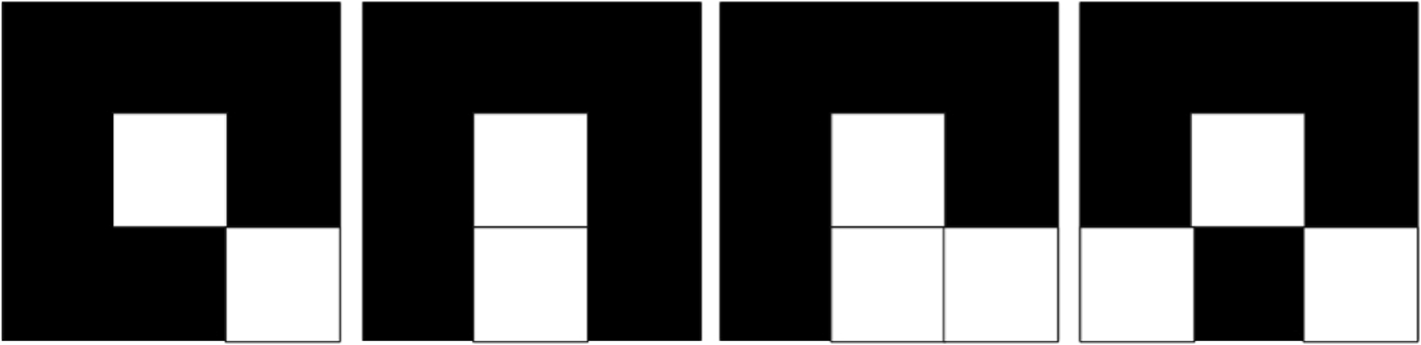
Total four types of 3 × 3 endpoint grids. (Two and three endpoints)

Next, to make the detected edges as complete and robust as possible, here we present how we mend the neglected part of an edge. We use a 3 × 3 grid as an example, when the end points of an edge are examined in a 3 × 3 grid of pixels, four types of endpoints can be identified, as shown in Fig. 2.

**Fig. 3:**
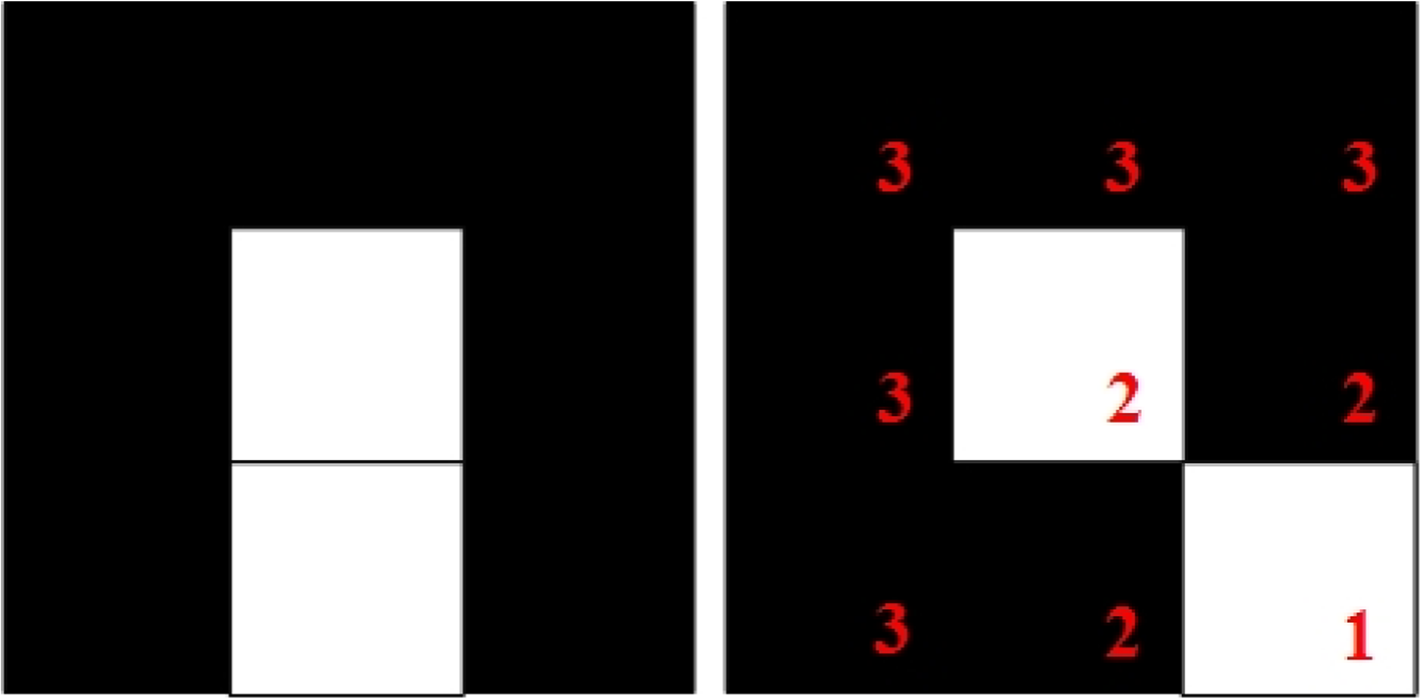
Grids with exactly one pixel connected to the edge endpoint

**Fig. 4:**
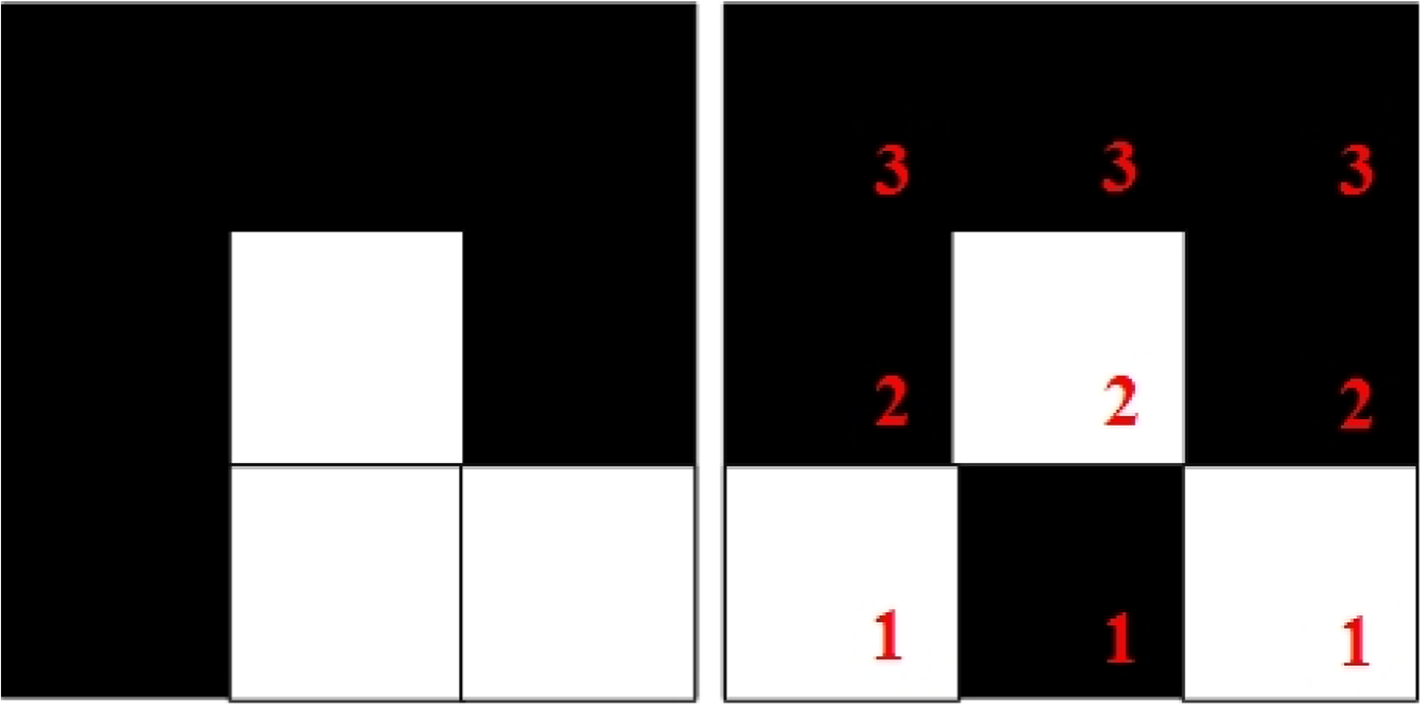
Grids with two pixels connected to the edge endpoint

These matrices are called boundary matrices. The white pixels in a boundary matrix represent the detected edges, while the remaining black pixels represent the background. Additionally, the white pixel in the center of the matrix represents an end point of the edge, which is extended by dividing the boundary matrix into one of the following two types: (1) Exactly one pixel is connected to the edge endpoint (Fig. 3), or (2) Two pixels are connected to the edge endpoint (Fig. 4).

Notably, both Figs. 3 and 4 showcase several pixels that are labeled with red numerals. Regardless of whether a boundary matrix is of Type 1 or Type 2, the method seeks a pixel marked with the numeral 3 to connect with a pixel marked with the numeral 2. The boundary matrix can then be divided into two unconnected regions. The pixel that is chosen to connect to a pixel marked with 3 is called the extension point, which is selected according to the difference between the average gray levels of the two nonconnected regions. The point that can make the largest difference is the extension point, and its pixel is set to white to mend the edges of a wound.

Despite this process, new extension points continue to be sought by making the previous extension point the center of a new boundary matrix. The procedure recurses until the average gray level value difference between the two nonconnected regions is smaller than a preset threshold value. The neglected portion of an edge is gradually filled when this process ends, and the subsequent detected edges of the image are all identified as strong edges.

**Fig. 5:**
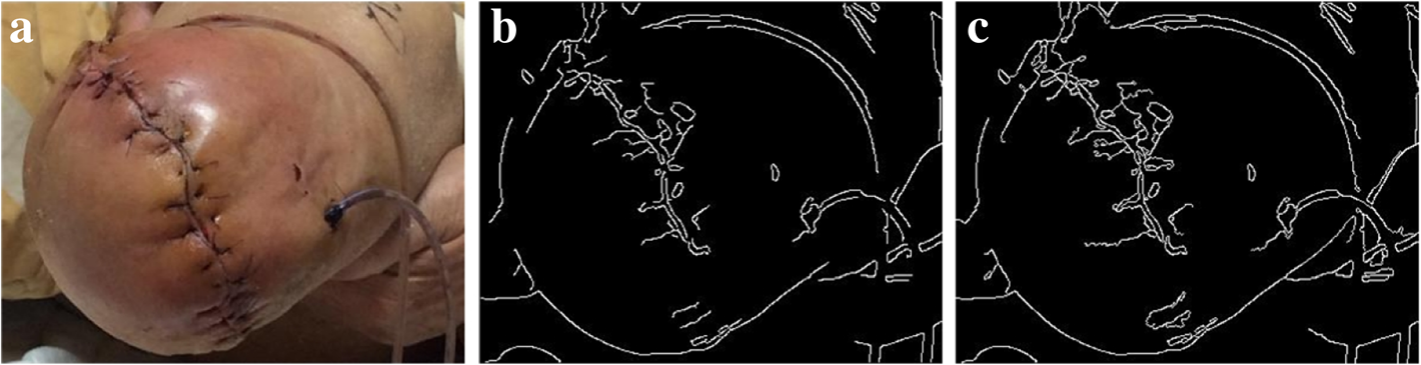
From left to right: **a** Original image; **b** Canny method applied to the image; **c** Edge-enforced results of the Canny method

**Fig. 6:**
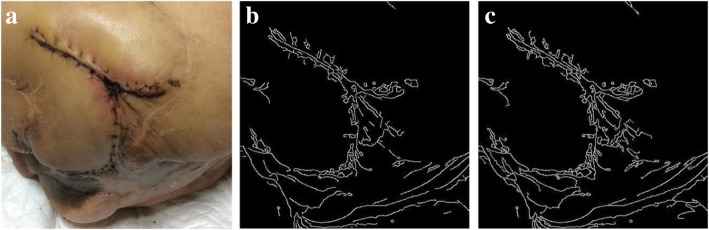
From left to right: **a** Original image; **b** Canny method applied to the image; **c** Edge-enforced results of the Canny method

Figures 5 and 6 showcase the results of two wound images after performing the edge enforcement algorithm.

##### Skin area demarcation

Due to the fact that images taken under various lighting conditions would have distinctive color biases, so different images captured on the same wound area may present biased skin colors. Thus, we propose the following steps to obtain the appropriate skin color value for each wound image:

- ***Step 1: Delimit regions***

A set of strong edges from a wound image is considered. Connected-component labeling (CCL), which is a method for identifying each object in a binary image is applied to group the detected edges. Adjacent edges are grouped into the same edge set; different edge set constitutes the edge set group as GE. Each edge set of GE can form a region, which contains all edges within the given edge set. If the starting image contains multiple edge sets, the image is divided into several nonconnected regions, which constitute a collection as RG.

- ***Step 2: Exclude non skin color area pixels***

As [20] reported, HSV (Hue, Saturation, Lightness) color space can describe skin color values. For example, in ethnically Asian and Caucasian subjects, the H channel is characterized by values between 0 and 50, and the S channel has values from 0.23 to 0.68. Because of our experimental results herein, the HSV skin color values are defined by the following ranges:

$$ {\mathrm{SH}}_{\mathrm{lbpt}\left(\mathrm{i}\right)}\geqq 0.035\&{\mathrm{SH}}_{\mathrm{lbpt}\left(\mathrm{i}\right)}\leqq 0.7\&{\mathrm{SS}}_{\mathrm{lbpt}\left(\mathrm{i}\right)}\geqq 0.005\&{\mathrm{SS}}_{\mathrm{lbpt}\left(\mathrm{i}\right)}\leqq 0.8\&{\mathrm{SV}}_{\mathrm{lbpt}\left(\mathrm{i}\right)}\geqq 0.35\&{\mathrm{SV}}_{\mathrm{lbpt}\left(\mathrm{i}\right)}\leqq 0.9, $$

where:

1 $$ {\displaystyle \begin{array}{l}{\mathrm{SH}}_{\mathrm{lbpt}\left(\mathrm{i}\right)}=\frac{\sum \sum \mathrm{imgzh}\left(\mathrm{lbpt}\left(\mathrm{i}\right)\right)}{\mathrm{size}\left(\mathrm{lbpt}\left(\mathrm{i}\right)\right)}\\ {}{\mathrm{SS}}_{\mathrm{lbpt}\left(\mathrm{i}\right)}=\frac{\sum \sum \mathrm{imgzs}\left(\mathrm{lbpt}\left(\mathrm{i}\right)\right)}{\mathrm{size}\left(\mathrm{lbpt}\left(\mathrm{i}\right)\right)}\\ {}{\mathrm{SV}}_{\mathrm{lbpt}\left(\mathrm{i}\right)}=\frac{\sum \sum \mathrm{imgzv}\left(\mathrm{lbpt}\left(\mathrm{i}\right)\right)}{\mathrm{size}\left(\mathrm{lbpt}\left(\mathrm{i}\right)\right)}\end{array}} $$

and *lbpt(i)* represents the labeled points for the ith nonconnected region in RG, imgzh, imgzs, imgzv represent the H, S, V color-space of the edge-enforced image. Subsequently, the regions with HSV values that are not in the range of skin color are filtered out and form a new collection as RG’.

- ***Step 3: Calculate the appropriate skin color values***

This paper selects the region that contains the most skin color pixels as the reference region from RG’, and calculates the optimum skin color values according to Eq. (2):

2 $$ {\displaystyle \begin{array}{l}{\mathrm{SH}}_{\mathrm{optm}}=\frac{\sum \sum \mathrm{imgzh}\left(\mathrm{optm}\right)}{\mathrm{size}\left(\mathrm{optm}\right)},\\ {}{\mathrm{SS}}_{\mathrm{optm}}=\frac{\sum \sum \mathrm{imgzs}\left(\mathrm{optm}\right)}{\mathrm{size}\left(\mathrm{optm}\right)},\\ {}{\mathrm{SV}}_{\mathrm{optm}}=\frac{\sum \sum \mathrm{imgzv}\left(\mathrm{optm}\right)}{\mathrm{size}\left(\mathrm{optm}\right)},\end{array}} $$

where *optm* is the labeled points for the region that contains the most skin color pixels. Notably, the skin color values calculated through Eq. (2) represent the general color for the skin area of the captured image.

- ***Step 4: Build the skin area image***

Finally, the presence value of each region for RG’ is determined according to the following procedure:
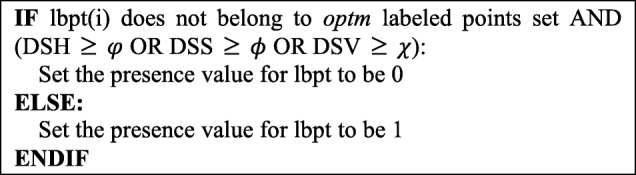


where: *φ* (e.g. 0.25); *ϕ* (e.g. 0.25); *χ* is (e.g. 0.2); is calculated according to the most probable difference between skin and non-skin region. *DSH, DSS, DSV* are the absolute values for (SH_optm_-SH_lbpt(i)_), (SS_optm_-SS_lbpt(i)_) and (SV_optm_-SV_lbpt(i)_). *lbpt(i)* represents the labeled points for the ith nonconnected region in RG’.

**Fig. 7:**
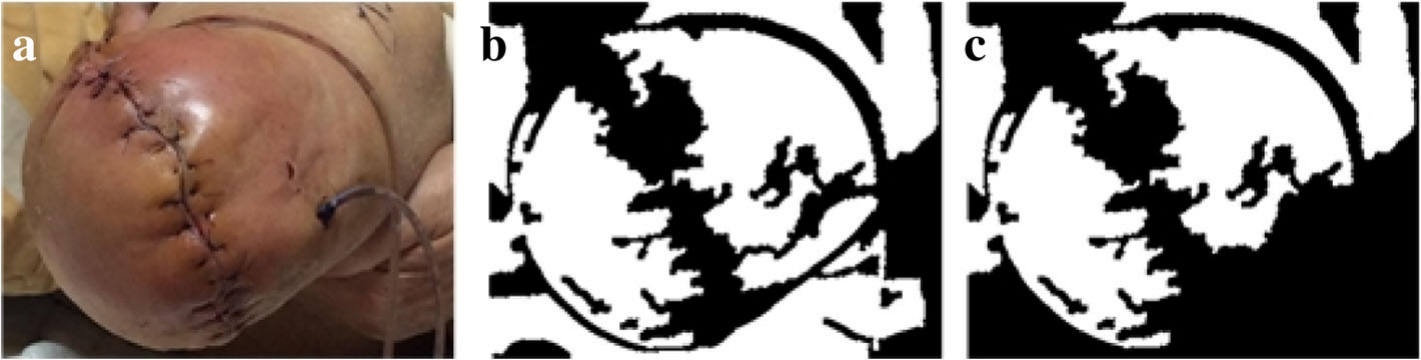
From left to right: **a** Original image; **b** Binary image composed of CC calculated connected regions; **c** Result image after filtering out zero presence value regions

The region with a zero presence value for RG’ will be treated as non-skin area so will be excluded. The pixels within the region with presence value one represent the possible skin area, as depicted in Fig. 7c. Once this skin area image is established, we will then conduct the corresponding process to delineate the wound region for this image.

##### Delineate the wound area

From our observation, the detected edge sets after applying Canny edge detector (CED) on the result image in the previous step contain not only the edge sets that can determine the wound region, but also a number of broken, as well as redundant edge sets formed by skin wrinkles.

In order to minimize the effect caused by these skin wrinkles for further analysis. This paper proposes below two steps to optimize the Canny threshold value through adjusting the value to find a stable number of edges:

- ***Step 1: Calculate Candidate Threshold***

This step begins by gradually incrementing the threshold value. Notably, when a higher threshold value is set, the number of lines detected is reduced, but the prominent contours remain unaffected. This step then check the edges with similar slope to avoid disruptions caused by wrinkles.

First, the detected edges are linked together through connected-component (CC) calculation to form a set of edge contours which is labeled GA. Second, the threshold value is increased, the edges are enforced and re-detected, then collected in a new set labeled E. Because the threshold is higher, the number of elements in E must be smaller than the initial edge set. Subsequently, the adjacent edges in set E are connected through CC calculation to form a new set of edge contours, which is labeled GB. Any elements in GA that are also in GB are then removed from GA and the result is defined as GC.

If there exists a number of edges with similar slopes for one edge counter in GC greater than the limit value, and the threshold value is less than the upper bound, then increment the threshold value and repeats the previous procedure.

**Fig. 8:**
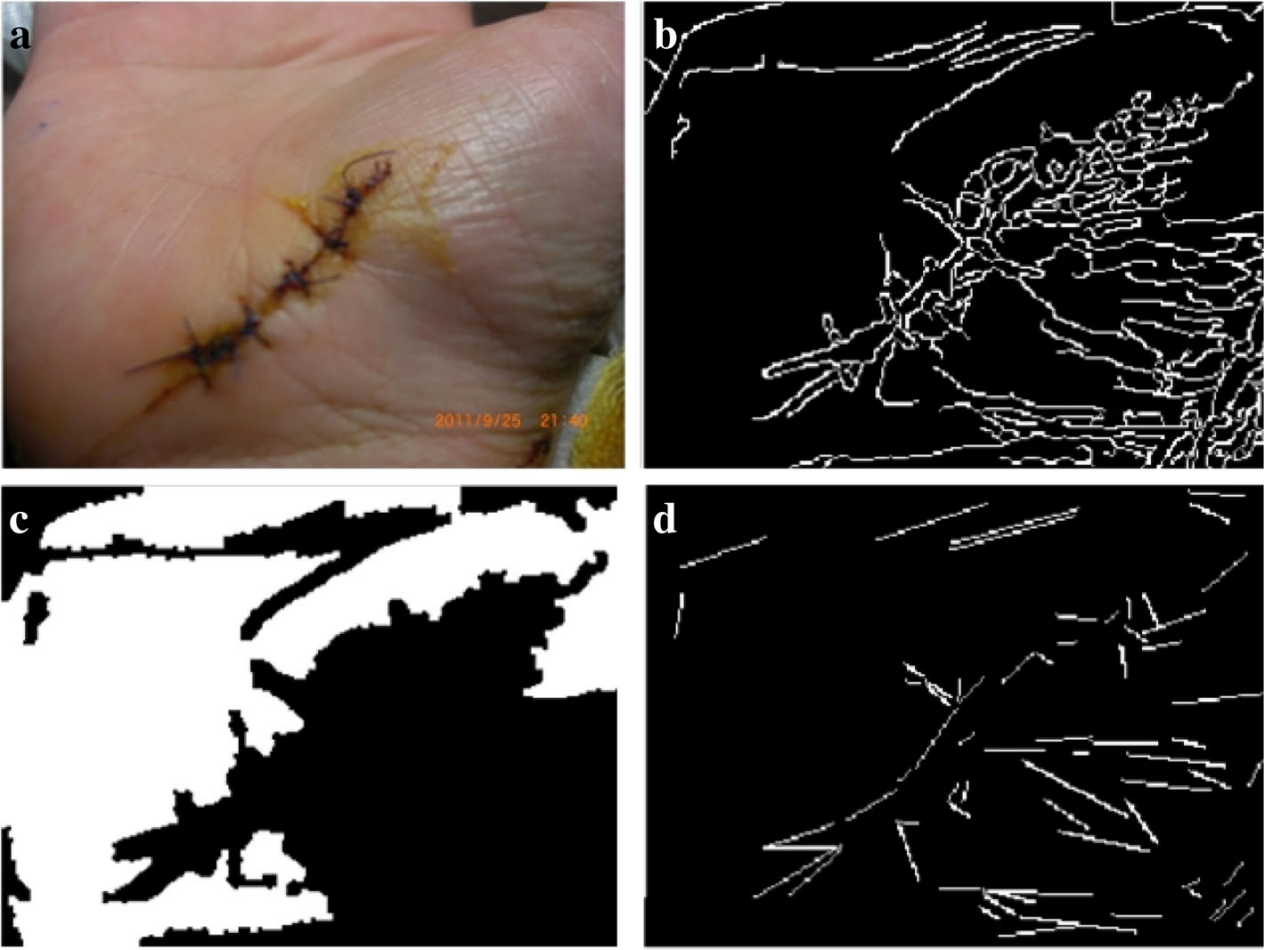
**a** Original image; **b** Edge-detected image; **c** Segmented image; **d** Slope lines image

Figure 8a shows an original palm image. Figure 8b presents an edge detection result that is severely impaired by the wrinkles of the palm; the lines in the bottom right of the figure are too complex. Figure 8c shows the result of segmentation given the edge contours from Fig. 8b. It is obvious that the cut area for the palm is not complete; specifically, the lower right part of the palm is missing. Figure 8d presents the result of wrinkle detection. By linking the end points of each wrinkle, we derive a set of straight lines; these line sets are then organized by the slopes of the lines in proximity area.

**Fig. 9:**
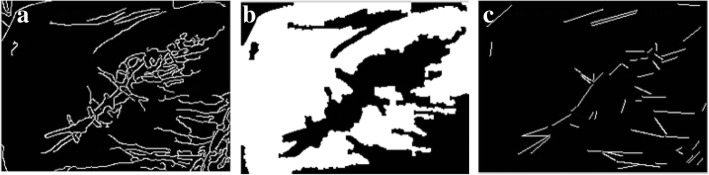
From left to right: **a** Edge-detected image; **b** Segmented image; **c** Slope lines image

**Fig. 10:**
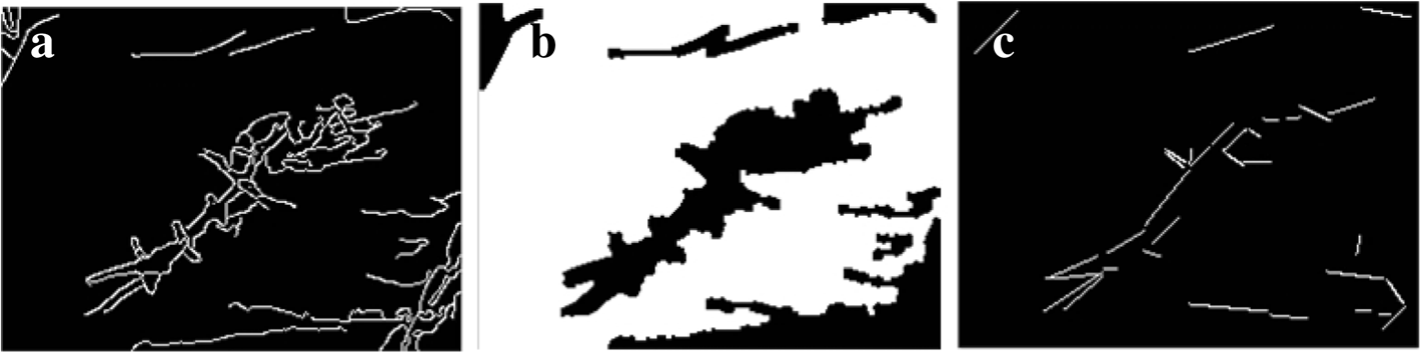
From left to right: **a** Edge-detected image; **b** Segmented image; **c** Slope lines image

Similarly, Fig. 9a shows an edge-detected image with a higher threshold value, while Fig. 9b shows the result of segmentation given the edge contours from Fig. 9a. Figure 9c exhibits the straight lines that are produced by the wrinkles. A third set of figures (Fig. 10a, b, and c) demonstrate similar results, although with a higher threshold value than that of Fig. 9.

The segmentation results for skin-colored regions of the palm image in Figs. 9b and 10b are more complete than the results offered in Fig. 8c. Therefore, the system can automatically correct the segmentation results to make the palm image more complete. The threshold value obtained in the above step is called the Candidate Threshold, and is calculated through the following CAN_THRESHOLD algorithm:
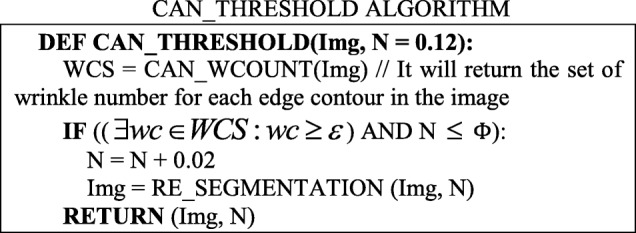


The *ε* value (e.g. 20) and the Φ value (currently set as 0.26) are calculated from the wound data sets composed of 159 surgery wound images. The Φ value is an upper-bound value and can be adjusted in the future.

- ***Step 2: Calculate Optimized Threshold***

Notably, the Candidate Threshold obtained in the aforementioned steps may cause the CED to detect too few edges, and thus may remove excess skin area around the wound region. These are adverse to subsequent analytical steps such as swelling or infection analysis of the wound. Furthermore, we must also consider the impact of skin wrinkles when revising the threshold values downward.

The steps to optimize the threshold value begins by defining the Candidate Threshold as the base reference value. First, CCL links adjacent edges to form a set of edge contours termed GA’. The Candidate Threshold value is then reduced and the image is analyzed to produce a new edge set named E’. The CC procedure links the adjacent edges in E’ and forms the set of edge contours termed GB’. The subset of elements in GB’ that is not within the intersection of GA’ and GB’ is named GC’. Next, the average RGB value for GC’ is calculated; however, if this value is too close to the average RGB value for pixels outside GB’ and the threshold is greater than the lower limit, then decrements the threshold value and repeats the previous steps.

**Fig. 11:**
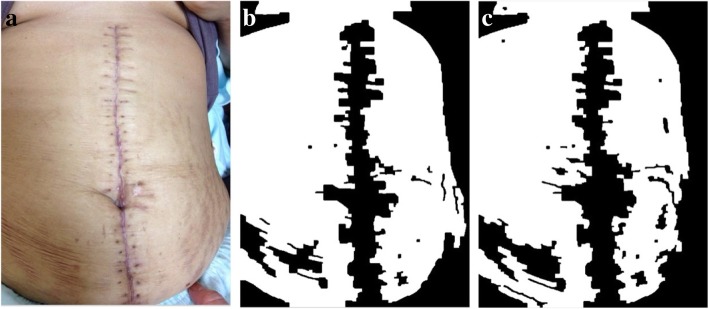
From left to right: **a** Original image; **b** Original segmented image; **c** Lowered threshold image

**Fig. 12:**
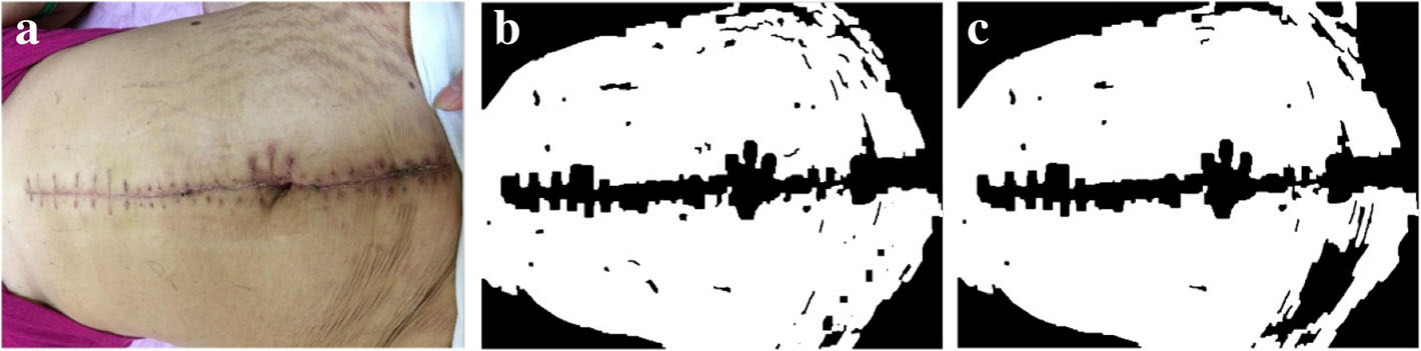
From left to right: **a** Original image; **b** Original segmented image; **c** Lowered threshold image

Ocassionally, pixels depicting wrinkles can cause incorrect segmentation when the Candidate Threshold is lowered, which leads to the errors demonstrated by Figs. 11 and 12.

Therefore, to minimize errors, this method will also judge the number of wrinkles and related effects in the image. When the system lowers the threshold value, for each edge contour in GC’, it will also check whether the number of edges with similar slope values is greater than the upper limit value; if this occurs, these edges are ignored when calculating the average RGB values. Thus, the Candidate Threshold obtained in this step is called the Optimized Threshold, and is calculated through the following OPT_THRESHOLD algorithm:
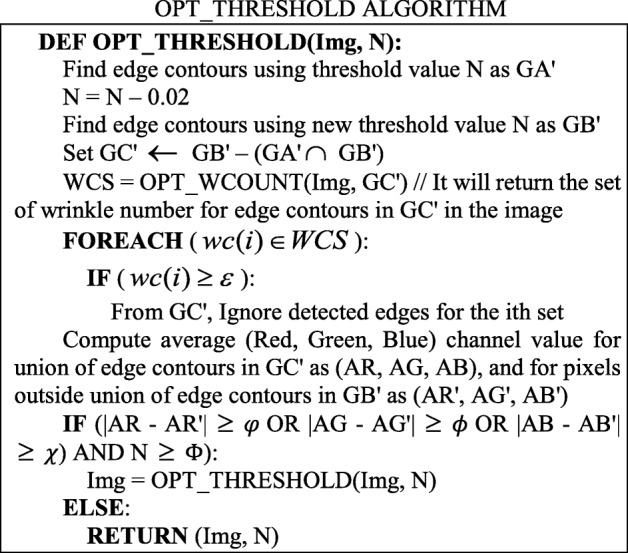


The *ε* value (e.g. 40), *φ* value (e.g. 38), *ϕ* value (e.g. 40) and *χ* value (e.g. 40) and Φ value (currently set as 0.06) are calculated from the same wound data sets composed of 159 surgery wound images. The Φ value is a lower-bound value and can be adjusted in the future.

By applying the Optimized Threshold to the CED, this system can detect the edge sets that are optimal for the subsequent analysis steps and retain the appropriate skin areas around the wound; these edge sets are labeled as AE.

##### Reconstruct the wound area

**Fig. 13:**
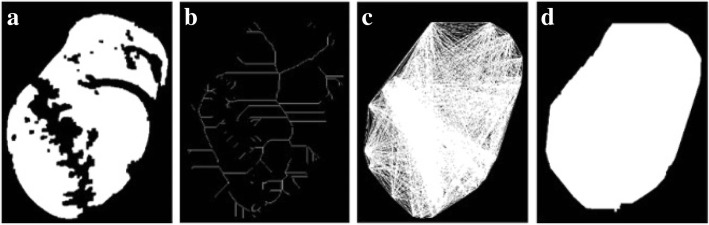
From left to right: **a** Input image; **b** Image after applying morphology topological skeleton and Hough transformation; **c** Image after connecting end points; **d** Final coverage area

From our observation, the wound area must include some of the edges from AE, so it is a subregion which contains all of the edges from AE. Thus, this paper proposes a method to reconstruct the wound area by discovering its topological skeleton within the edge sets from AE to form the backbone of the wound area for the original image, as outlined in Fig. 13.

**Fig. 14:**
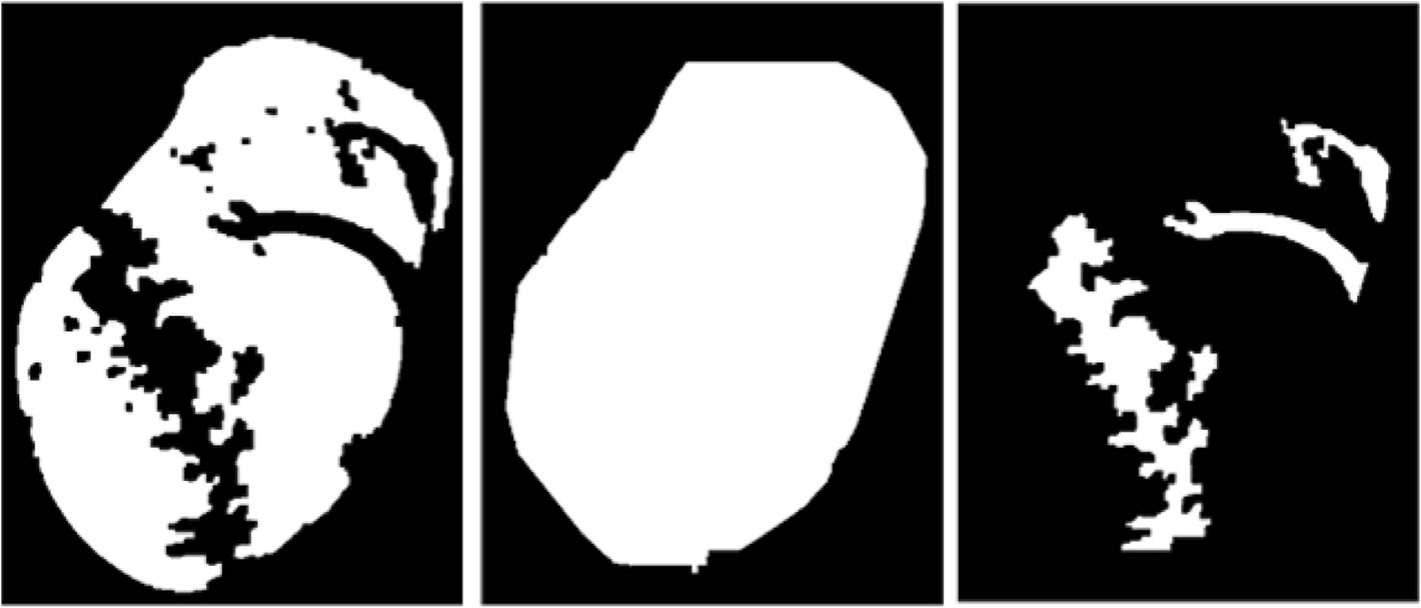
Final wound area image obtained after performing complementary calculation

Two inputs, namely the full image containing the wound and nonwound area, and the image of the preceding CED, are entered into a complementary calculation that produces an image of the wound area subregion. An example output is presented in Fig. 14. Through above steps, finally the wound area are designated and the non-wounded area are suppressed.

To compare the computer segmented results with physician manual segmented results. We adopt the boundary-based evaluation method from [21]. Wound coverage rate and exceeded rate are calculated through Eq. (3):

3 $$ {\displaystyle \begin{array}{l} CoverageRate=\frac{\left(N1- CN\right)}{N1}\\ {} ExceedRate=\frac{CP}{N1}\end{array}} $$

Where N1 is the manual segmented region. CP is the exceeded region for computer segmentation minus manual segmentation (positive parts). On the contrary, CN is the negative parts. Table 1 summarizes the evaluated results and coverage rate for total 100 segmented images with gold standard set up by three physicians. Overall, it achieves 76.44% true positive rate and 89.04% accuracy value, our segmented method can retain all necessary region compared to the manual segmented results from three physicians. The exceeded region can be used to monitor the wound healing process in the surrounding skin area. From our observation, there are inter-difference between the agreement of the manual segmented results even among three professional physicians.

**Table 1:** Calculated results from 100 samples & wound area coverage rate analysis

Statistics		Coverage Rate	No. (Rate)
TPR	76.44%	> 90%	46(46%)
ACC	89.04%	80% ~ 90%	12(12%)
SPC	91.58%	70% ~ 80%	9(9%)
PPV	63.85%	60% ~ 70%	9(9%)
Wound area coverage rate	76.44%	50% ~ 60%	5(5%)
		< 50%	19(19%)
Total			100

**Fig. 15:**
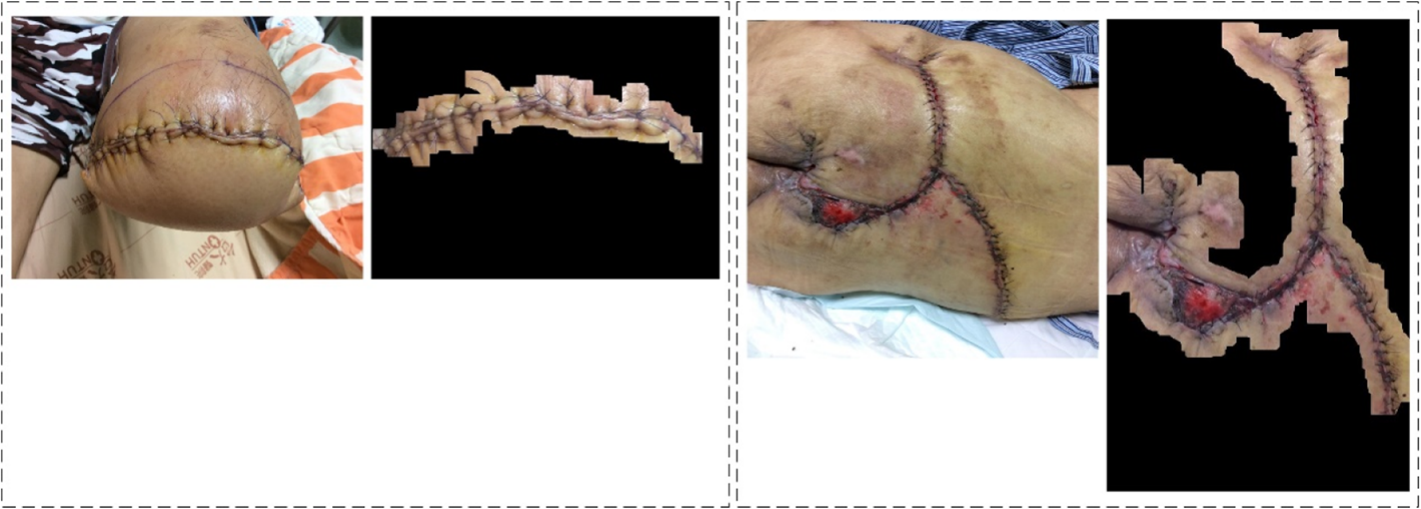
Two examples of original wound image and segmented results after performing robust wound image segmentation

**Fig. 16:**
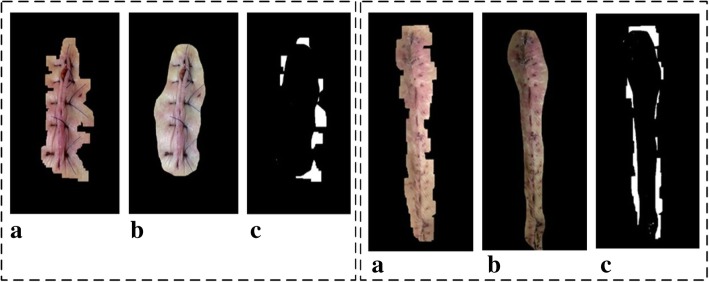
Two example comparisons between Robust Wound Segmentation and manual segmentated results from the agreement of three physicians. In each set from left to right: **a** Robust Wound Segmentation results; **b** Manual segmented results; **c** Difference between two results. Wound coverage rate for left data set is 83.07%; whereas for right data set is 92.45%

**Fig. 17:**
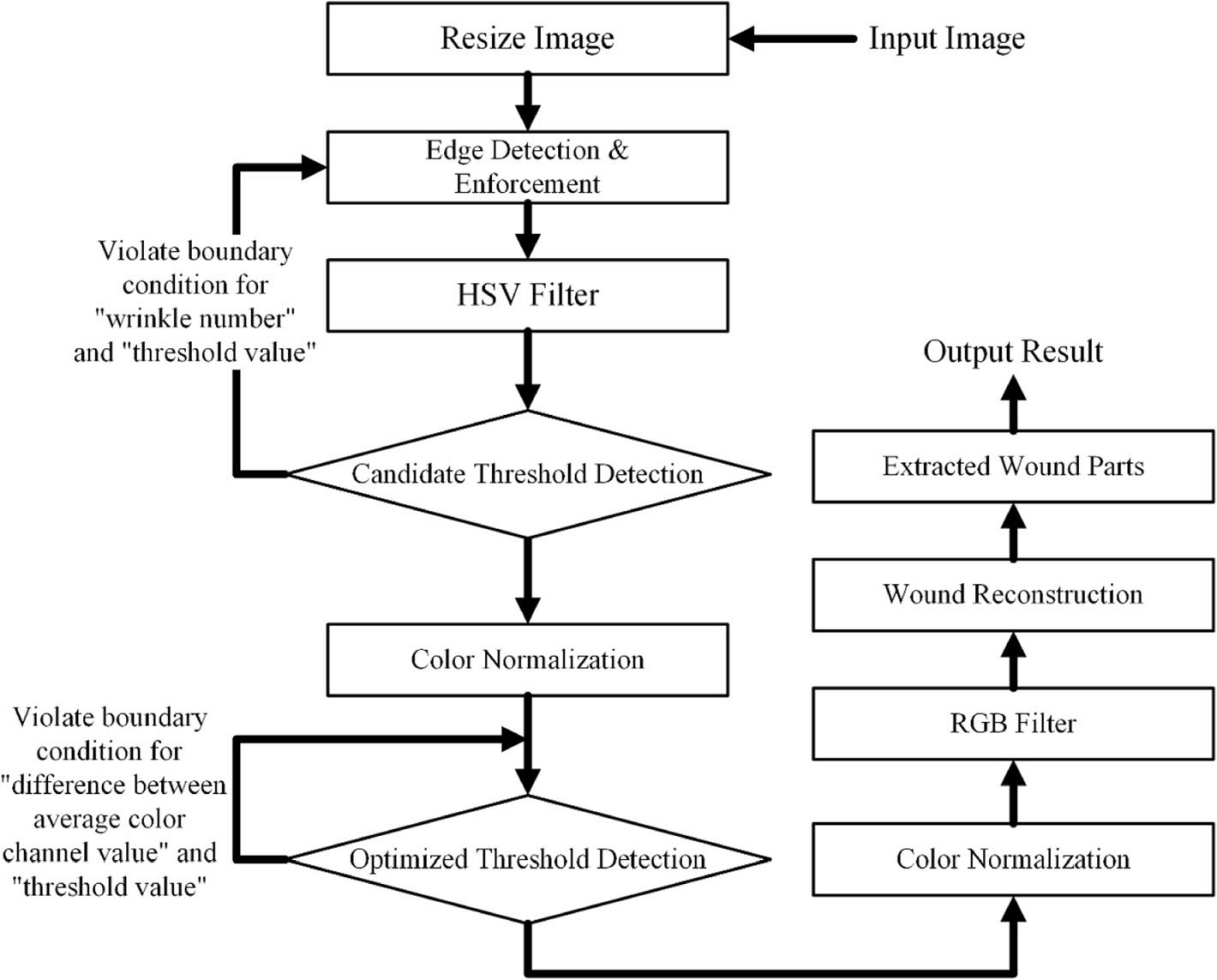
Flow chart for proposed Robust Wound Segmentation

Table 2 presents a summary of the statistics for the robust image segmentation method, completeness is judged by three physicians which means the segmented result can represent the whole wound area for infection analysis. Figure 15 gives two cases after robust image segmentation is performed. In both cases, this process completely removes the noisy background information but retaines the clinically noteworthy skin area near the wound region for infection analysis. Figure 16 compares two cases for computer segmented and manual segmented results. Figure 17 is the complete flow chart for Robust Wound Image Segmentation method.

**Table 2:** Segmentation results for different sites

Wound site	Number of cases	Completely segmented	Incompletely segmented
Face	3	1	2
Chest	47	44	3
Abdomen	23	21	2
Back	11	10	1
Hand	7	4	3
Podiatry	9	7	2
Total	100	87 (87%)	13 (13%)

### SVM-based wound analysis interpretation

From our literature review, recent works for wound interpretation and healing assessment include: Wannous et al. [5] apply J-SEG to do wound segmentation and classification based on color descriptors such as mean color descriptor (MCD), locally adapted dominant color descriptors (DCD) with 2-D and 3-D color histograms to do wound assessment; Veredas et al. [17] apply neural networks and Bayesian classifiers to do wound tissue characterization for 113 wound region images. It achieves 78.7% sensitivity, 94.7% specificity and 91.5% accuracy; Loizou et al. [22] apply Snake to do image segmentation and use texture feature to do wound healing assessment for 40 images from 10 cases.

For chronic wound healing, in order to do early infectin detection, our method must examine each region for a specific wound area carefully and throughly. Therefore, there is a need to develop a wound assesement method which can detect correct ROIs for a wound area. Furthermore, it can then examine each ROIs completely and thoroughly. Here we introduce a SVM-Based wound infection interpretation method to solve this type of problem. The segmentation results from the Robust Wound Segmentation method will be used in this part.

#### Position wound suture site

The feature points located in the wound suture site that are capable of expressing the wound position information are detected through the following procedure:

##### Highlight the wound area

First, the original image is converted to a grayscale image according to Eq. (4):

4 $$ Y\leftarrow 0.299\cdot R+0.587\cdot G+0.114\cdot B $$

where *Y* is the computed gray scale value for the given red, green, and blue color channels of the original wound image.

To highlight the contrast between wound suture sites and surrounding skin areas, Otsu’s adaptive thresholding [23] algorithm is adopted to convert the grayscale image into the binary image. Subsequently, the wound area is notably more visible in this binary image.

##### Highlight the wound characteristics

To detect the feature points in wound suture site, this method firstly considers scale-invariant feature transform (SIFT) because SIFT features can effectively be applied to tasks that require identification of matching locations in the images. SIFT was proposed by David Lowe in 1999 [24]. The algorithm uses Difference of Gaussians (DoG), which is an approximation of the Laplacian of Gaussian (LoG) methods. DoG is obtained as the difference of Gaussian blurring of an image at different scaling parameters.

Besides SIFT features, cross-shaped features are considered too. This is because from our observation, most suture wounds are stitched perpendicular to the orientation of the main incision. Thus, cross-shaped features can also be used to identify the location of the wound suture sites.

**Fig. 18:**
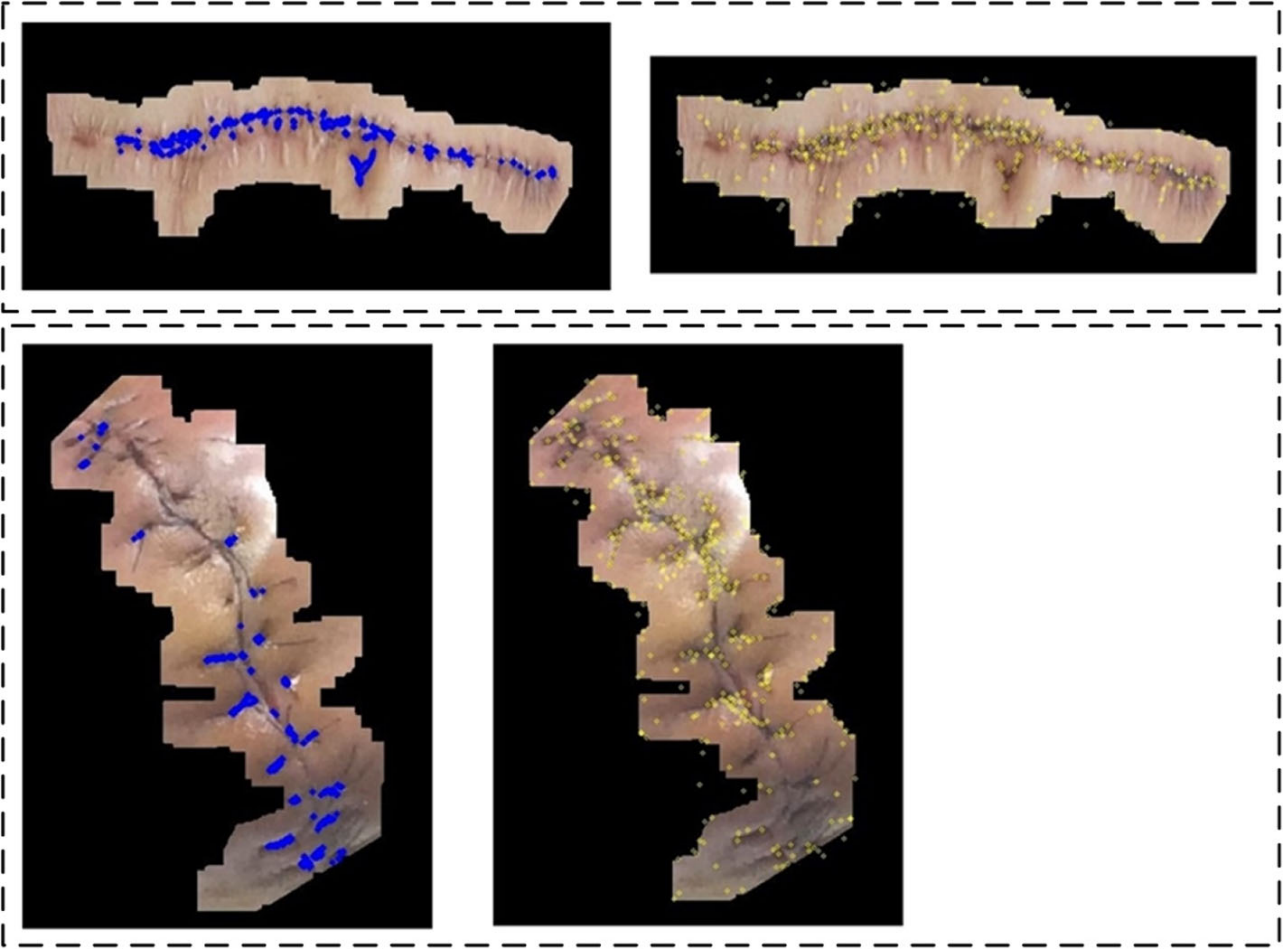
Two example results after applying cross-shaped (Left) and SIFT (Right) features

Figure 18 showcases two examples of the feature points detected for cross-shaped (Blue dotted) and SIFT features (Yellow dotted). Upon examining the comparison results from our testing datasets, we decide to apply the cross-shaped features in the future steps because it can find suitable feature points while keeping low noise level from non suture sites.

To detect the cross-shaped features, we apply a morphology shape method on the wound image to extract the pixels located in the cross-shaped regions. The core for the morphology shape method is to define the structuring element. Therefore, a 5 × 5 cross-shaped structuring element is defined in Eq. (5):

5 $$ \mathrm{Cross}\ \mathrm{Kernel}=\left[\begin{array}{ccccc}0& 0& 1& 0& 0\\ {}0& 0& 1& 0& 0\\ {}1& 1& 1& 1& 1\\ {}0& 0& 1& 0& 0\\ {}0& 0& 1& 0& 0\end{array}\right] $$

The implementations for various Morphological functions can be attributed to the combination of erosion and dilation operations. Therefore, our method conducts erosion and dilation on the binary image pixels according to the cross kernel defined. Subsequently, the pixels retained in the image are all pixels located in the cross-shaped regions.

##### Extract feature points

Apparently, adjacent pixels may locate in the same cross-shaped area. The pixels retained in the cross-shaped regions can further be clustered to form the appropriate wound feature point. Therefore, CCL algorithm [25] is used to connect adjacent pixels, and then group these pixels into multiple clusters. Ultimately, each cluster produces a wound feature point, which can be used to calibrate the position of the wound suture site.

#### ROI detection

In this context, the ROI is the region of the wound suture site. When the feature points are extracted, they are grouped to form different ROIs through the following two steps.

##### Cluster

**Fig. 19:**
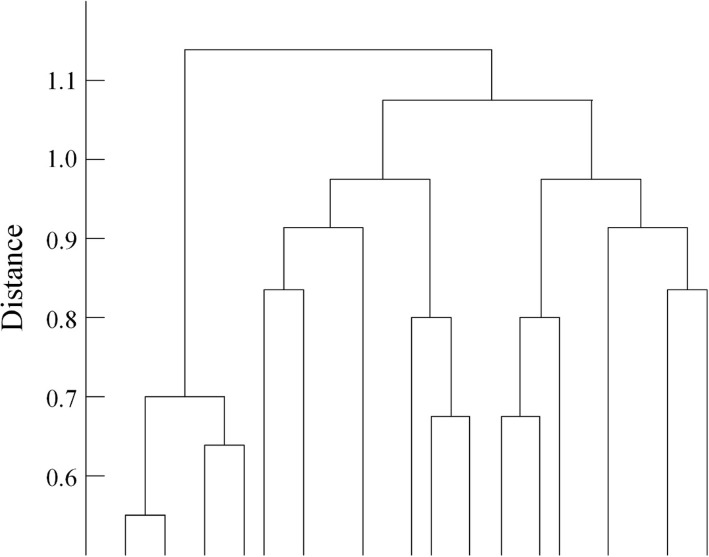
HCM to group the feature points into feature point sets. The unit of distance is measured by the distance between two adjacent pixels in the image

From our observation, the wound feature points are located in the wound-stitching site, and these feature points tend to be distributed in clusters. In order to locate each wound suture site, these extracted feature points are divided into multiple groups according to their distribution locations. We use Hierarchical clustering method (HCM) [26] to group these feature points into a hierarchy tree (Fig. 19).

Each hierarchy in the tree represents a clustering method for a different point’s number. Each clustering method divides these feature points into a plurality of feature point sets, and each set represents an individual wound suture site.

##### Determine optimal clustering number

i. *Calculate scores*

Various clustering methods will produce different number of detected wound suture sites. Therefore, we propose an optimal clustering method here that can determine the most appropriate number for wound analysis interpretation.

First, the clustering number score for each hierarchy in the tree are calculated according to Eq. (6):

6 $$ {\displaystyle \begin{array}{l}S(i)=\frac{\max \left(a(i),b(i)\right)}{b(i)-a(i)}\\ {}S(i)=\left\{\begin{array}{c}1-a(i)/b(i),\kern0.5em \mathrm{if}\ a(i)<b(i)\\ {}0\kern4.5em ,\kern0.5em \mathrm{if}\ a(i)=b(i)\\ {}b(i)/a(i)-1,\kern0.5em \mathrm{if}\ a(i)>b(i)\end{array}\right\}\end{array}} $$

where *S(i)* is the silhouette score (SC) for hierarchy *i*, defined in terms of *a(i)* and *b(i)*; *a(i)* is the average element in a cluster; *b(i)* is the number of clusters; and *i* is the index variable for *a* and *b*, which spans from 2 to (number of feature points / 2).

When the SC is maximal, the average element in a cluster is minimal and the number of clusters is maximal. This means that each feature point represents one wound region, and the number of feature points is equivalent to the number of wound regions.

**Fig. 20:**
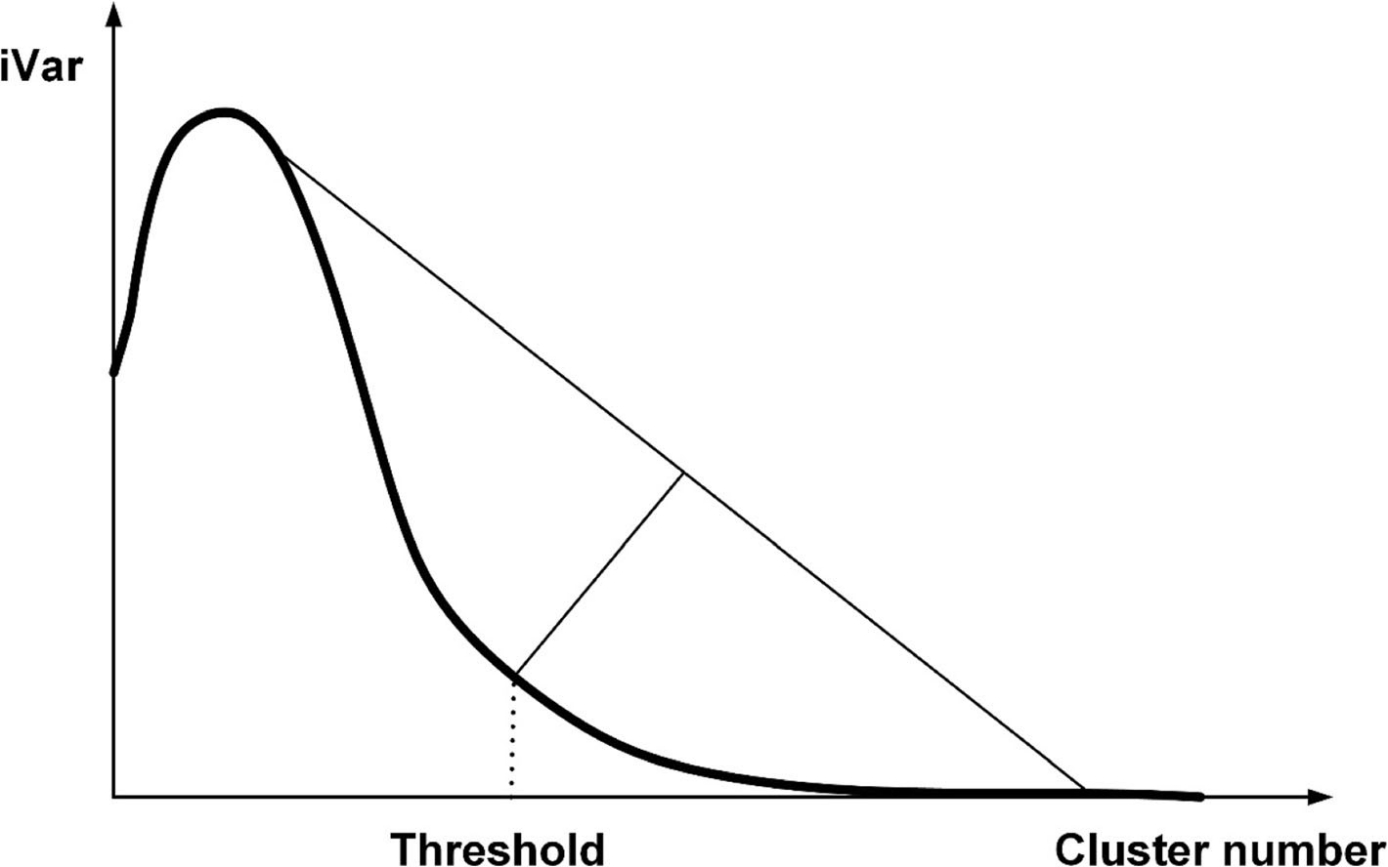
Horizontal axis represents the number of clusters; the vertical axis represents the iVar calculated for each hierarchy. The rate of decline of iVar gradually begins to converge at the marked threshold point

By considering the data from many wounds, we can draw a graph with the number of clusters on the horizontal axis and the corresponding SC on the vertical axis. Overall, as the number of clusters increases, the SC increases; however if the number of clusters increases too much, the system reaches convergence. From this observation, the SC is defined as Var, and thus iVar = 1 / Var. Figure 20 depicts the relationship between iVar and the corresponding number of clusters.

The iVar for an average element in a cluster is inversely proportional to the number of clusters, so an increase in the number of clusters corresponds with a decrease in the iVar. Thus, when the number of clusters notably increases, the iVar must rapidly decrease. Eventually, the rate at which iVar drops tapers to convergence as the number of elements within each cluster reaches the minimal level. Here, we propose a method to choose an appropriate cluster number to derive the best suitable wound region.

ii. *Decide the threshold value*

For iVar, the declining rate of the value gradually reaches convergence at the threshold point indicated in Fig. 19. Empirically, iVar is a unimodal function, so this research applies a unimodal thresholding (UT) algorithm [27–29] to locate the threshold point. The corresponding feature point set is the most appropriate.

iii. *Detect ROIs*

For each feature point set, the area containing the feature points of this set forms the ROI. Thus, this paper calculates the minimal upright bounding rectangle for the specified feature point set to be the ROI.

#### SVM-based approach to interpret and analyze the wound area

**Fig. 21:**
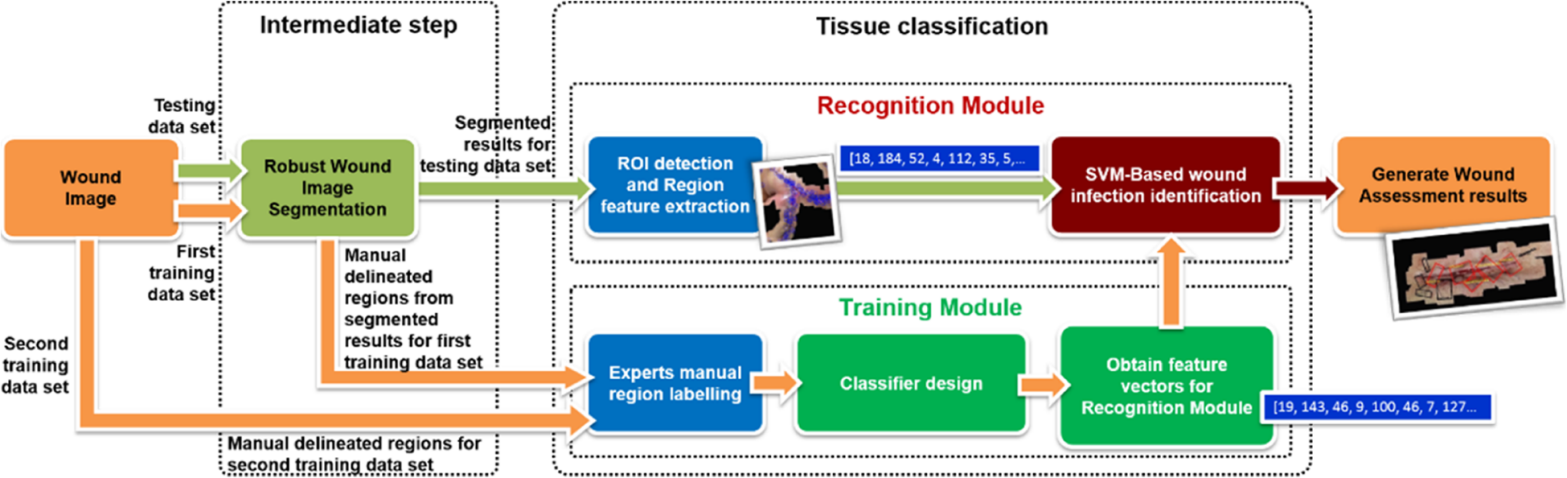
Complete flow for wound infection identification process

With reference to the CDC [30], the present research recognizes four wound infection classification types: (1) Clean, (2) Clean-Contaminated, (3) Contaminated, (4) Dirty or Infected. Proceeding from the CDC and clinical physicians’ judgment criteria, a SVM-Based wound assessment module are designed to detect the following four symptoms: (1) Swelling, (2) Granulation, (3) Infection, and (4) Tissue Necrosis. Specifically, for Swelling, this module can locate apparent swelling deformation characteristics along with signs of possible infection; For Granulation, this module can find any signs of bleeding or granulation; For Infection, this module can determine whether the wound area contains any signs of infection; For Tissue Necrosis, this module can detect any signs of bacterial infections on the wound area. Figure 21 illustrates our SVM-Based approach and its complete analysis flow. The following describes the steps necessary to establish this wound analysis module:

##### Establish sample training data sets for wound infection identifier

A postsurgery wound image may display a variety of wound types. This research selects and reviews a plurality of meaningful wound areas from the training data set composed of 159 general surgery wound images, then extracted an appropriate image size range to build the wound feature vectors for wound identification. The current training data set composed of 13 Granulation, 27 Infection, 62 Necrosis, 23 Swelling and 34 Normal cases. Some examples of our sample training data set used for identification of various different wound types are attached in the Figures 26, 27, 28, 29 and 30 in Appendix section. The collection of training data for the wound infection modules is ongoing, and the quantity of training data is steadily increasing. Thus, the accuracy of the entire prediction module is enhanced over time.

##### Calculate feature vectors

Feature vectors corresponding to each symptoms (Swelling, Granulation, Infection, and Tissue Necrosis) are designed. Because extensive training data sets are applied to calculate the feature vectors for each symptom, useful distinctions can be drawn regarding the diversity of wound infections displayed in images. The steps to calculate the feature vectors for the Swelling symptom are listed here:
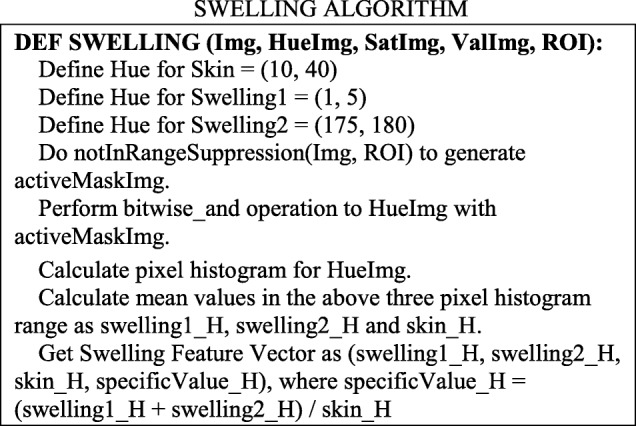


##### Train the wound infection identifier

Finally, the feature vectors in this paper are trained with a SVM [31, 32]. A polynomial kernel with degree three is used to train the wound identifier. The accuracy and computation time for tuning the hyperparameters of the SVM are listed in Table 3. The polynomial kernel then maps the original variables into a polynomial feature space, which enables the system to learn nonlinear models. As Table 3 reports, the results are computed within an acceptably short time by the polynomial kernel, and particularly with superior classification results compared with those of a linear kernel.

**Table 3:** Performance and computation time using different polynomial kernel degree

Kernel Degree	Accuracy For Anomaly Detection	Accuracy For Symptom Assessment	Computation Time
Degree = 1	84.32%	75.37%	1211 Mins
Degree = 2	85.07%	80.03%	232 Mins
Degree = 3	87.31%	83.58%	**32 M** **ins**
Degree = 4	83.58%	78.73%	35 Mins

Computation time was measured on an Intel Xeon E3 3.20 GHZ Quad Core CPU with 16GB RAM installed

Equation (7) reveals the polynomial kernel function used herein:

7 $$ K\left({x}_i,{x}_j\right)={\left(\gamma {x_i}^T{x}_j+r\right)}^d,\gamma >0 $$

where γ, *r*, and *d* are the kernel parameters, x_i_ and x_j_ are vectors in the input space (i.e., the vectors of features computed from training or test samples), *r* ≥ 0 is a free parameter trading off the influence of higher-order versus lower-order terms in the polynomial; and *d* is three (the degree of the polynomial).

## Results

**Fig. 22:**
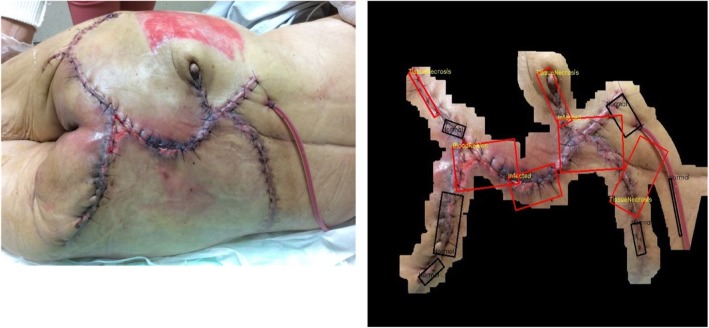
Analysis result for a very complex back surgery. There are total 12 ROIs detected. Six of them are interpreted as normal (Black), three of them are interpreted as necrosis (Red), two of them are interpreted as infected (Red), one of them is interpreted as granulation (Red)

**Fig. 23:**
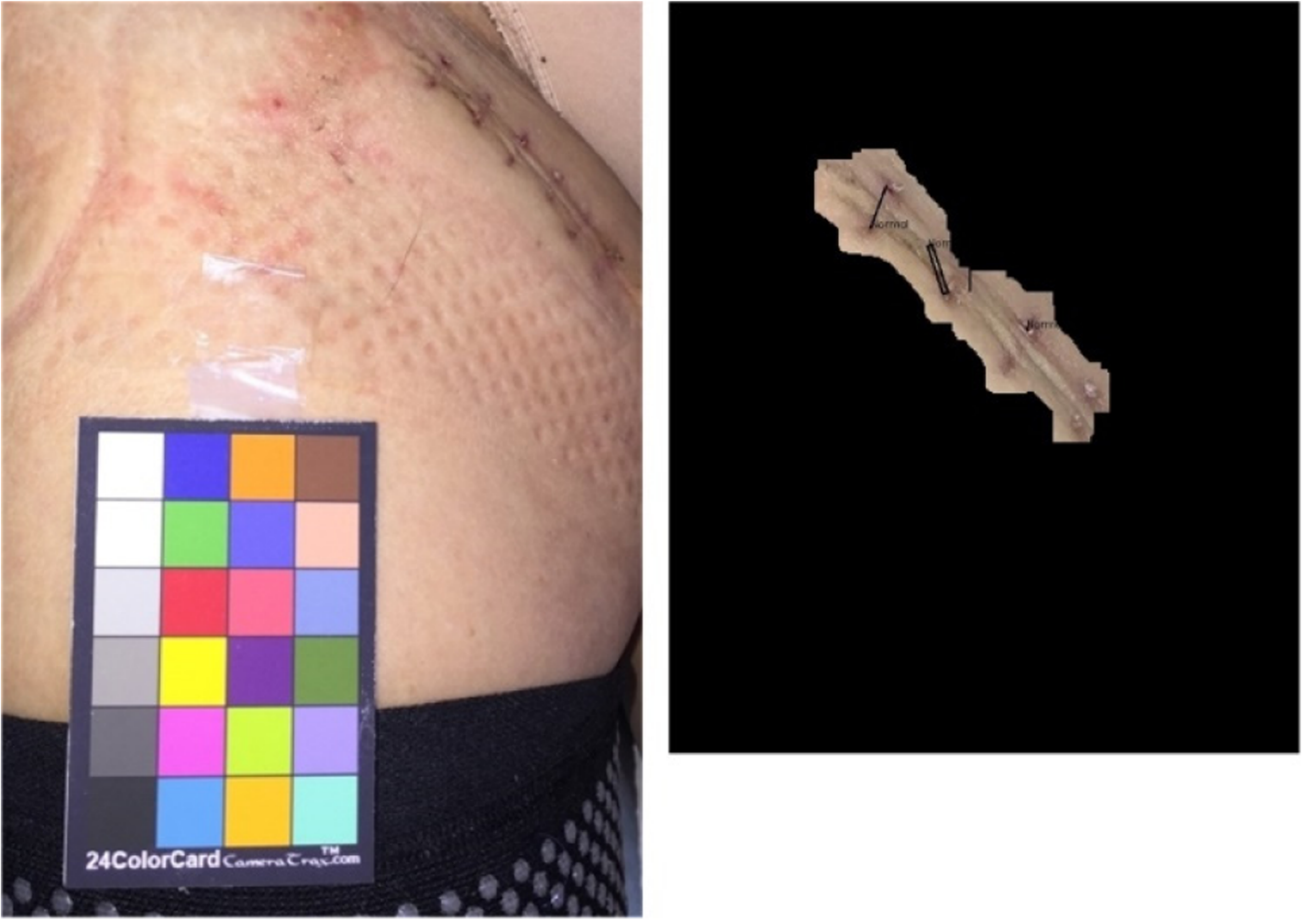
Analysis results for a non-infected sutured wound region. All of the three detected ROIs are interpreted as normal (Black)

**Fig. 24:**
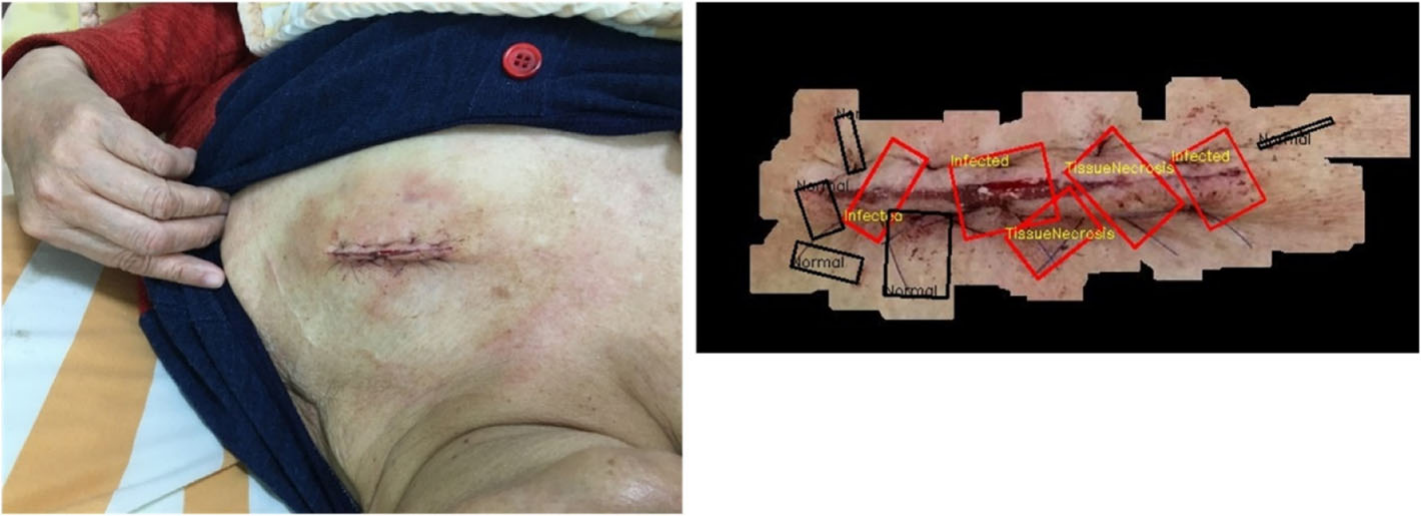
Analysis results for a patient who underwent a chest surgery. There are total 10 ROIs detected. Five of them are interpreted as normal (Black), two of them are interpreted as Necrosis (Red), three of them are interpreted as Infected (Red)

**Fig. 25:**
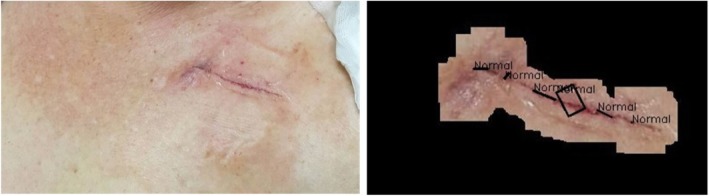
Analysis result for a patient who underwent a cardiac pacemaker surgery without wound infection. All six detected ROIs are interpreted as normal (Black)

**Fig. 26:**
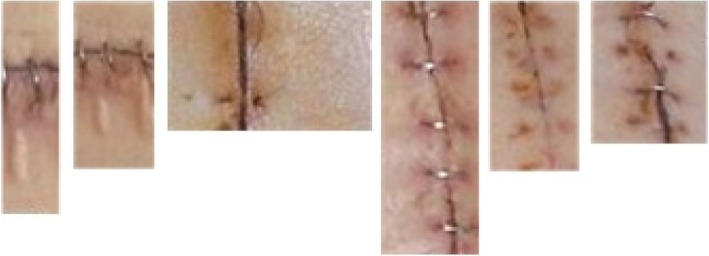
Normal non-infected sample data set

**Fig. 27:**
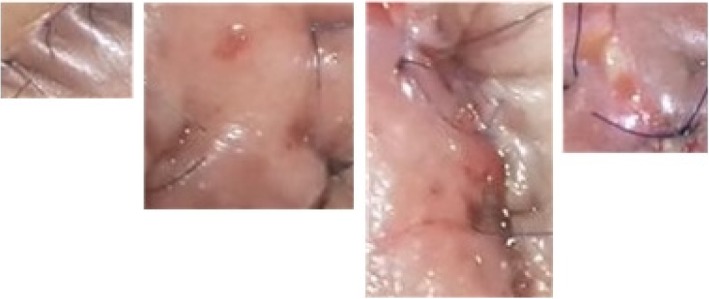
The swelling sample data set. In order to detect if there are any signs of swelling for the skin around wound area, or apparent swelling deformation characteristics along with signs of possible infection. This paper incorporates the wound swelling sample data set

**Fig. 28:**
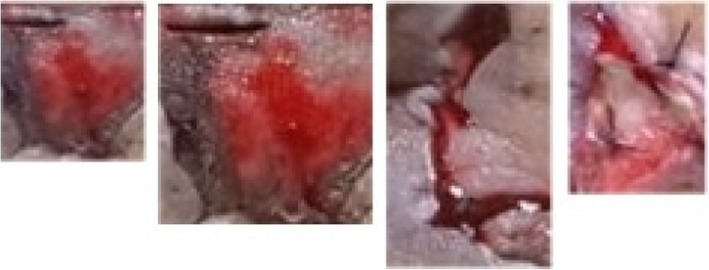
The granulation sample data set. The granulation sample data set is added to find out any signs of granulation occurred in the wound area. If the wound has any signs of granulation or bleeding, it requires immediate medical assistance intervention

**Fig. 29:**
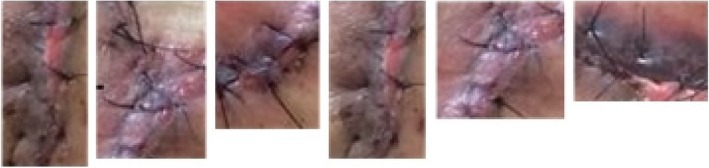
The infected sample data set

**Fig. 30:**
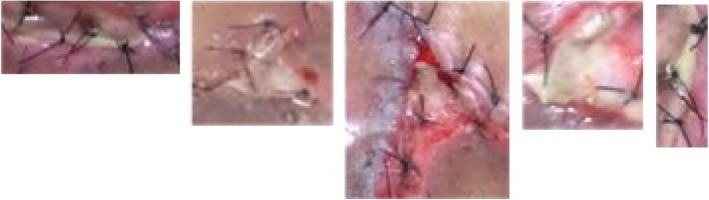
The tissue necrosis sample data set. In order to detect if there are any signs of bacterial infections, black necrotic tissue, pus, or other tissue necrosis signs for the wound area, this paper incorporates the necrotic tissue sample data set

In this research, 134 surgical wound sample images, including chest, abdomen, back, hand, and podiatry wounds, are processed by robust image segmentation and SVM-based wound infection assessment. The majority vote of three medical doctors is applied as a criterion standard to validate the capability of the proposed mechanism. Anomaly detection and Symptom assessment are carried out where Anomaly detection checks if the image is assessed as “Normal” or “Abnormal” with respect to its ground truth; Symptom assessment checks whether the symptom (Granulation, Infection, Necrosis, Swelling) is presented in the wound area. Each symptom will be examined in the wound area for *TP*, *TN*, *FP*, *FN*, so there will be 536 (=134*4) tests for symptom assessment. The results of *Accuracy*, *TP*, *TN*, *FP*, *FN* for Anomaly detection and Symptom assessment are presented in Table 4. And the detailed evaluaton results for each symptom assessment are presented in Table 5. Figure 22 depicts a very complex general surgery on back case wherein six of the 12 detected ROIs are regarded as potentially infected regions. Figure 23 shows a non-infected sutured wound case wherein all three detected ROIs are regarded as normal region. Figure 24 presents a complex cardiac pacemaker surgery wound region, the assessment result indicates that of the 10 detected ROIs, five of them are regarded as potentially infected regions. Finally, Fig. 25 showcases a rather simplified cardiac pacemaker surgery where all six detected ROIs are regarded as normal region.

**Table 4:** Performance for surgical data sets

Characteristics of dataset and algorithm	n or %
Anomaly Detection	
TP	95
TN	22
FP	6
FN	11
Accuracy	87.31%
Symptom Assessment	
TP	189
TN	259
FP	24
FN	64
Accuracy	83.58%

Symptom includes: Granulation, Infection, Necrosis, Swelling

Total samples: 134

**Table 5:** Performance evaluation for each symptom

	Granulation	Infection	Necrosis	Swelling
TP	19	44	77	49
TN	87	71	36	65
FP	8	2	5	9
FN	20	17	16	11
Accuracy	79.10%	85.82%	84.32%	85.07%

## Discussion

This augmentation mechanism was intentionally designed for clinical decision support, and it has been demonstrated reliable enough to reduce the need for face-to-face diagnoses. The performance of the SVM-Based wound infection assessment will continue to improve through the ongoing accumulation of training data sets. In the future, our research will focus on incorporating wound image texture feature information (including energy, entropy, and skew) into the feature vectors to improve the overall prediction performance. The work of using deep-learning technique for the wound infection assessment module is already on-going. Another work to use the standard 24 color card to determine the exact wound size and do color calibration is on-going too, this can give very precise statistical information for the wound area.

## Conclusion

In this paper, an algorithm to conduct edge enforcement, automatic threshold adjustment, and wound area reconstruction for robust wound image segmentation based on edge and color information is proposed. This algorithm can eliminate background noise while processing the relevant image data. Additionally, a SVM-Based wound assessment algorithm that calculates the positions of wound suture sites, and an optimal clustering method based on a unimodal Rosin thresholding algorithm to detect precise ROIs, together with feature vectors calcuted to be used for infection interpretation is proposed. To facilitate the use of this method and analytical framework, an automatic wound interpretation app and an accompanying website are developed.

## Abbreviations

CC: Connected component
CCL: Connected component labeling
CED: Canny edge detector
DoG: Difference of Gaussians
HCM: Hierarchical clustering method
IRB: Institutional Review Board
LoG: Laplacian of Gaussian
NTUH: National Taiwan University Hospital
ROI: Region of interest
SC: Silhouette score
SIFT: Scale-invariant feature transform
SSI: Surgical Site Infection
SVM: Support vector machine
UT: Unimodal thresholding
